# DeGenPrime provides robust primer design and optimization unlocking the biosphere

**DOI:** 10.1093/bioadv/vbae044

**Published:** 2024-03-14

**Authors:** Bryan Fulghum, Sophie H Tanker, Richard Allen White

**Affiliations:** Department of Bioinformatics and Genomics, North Carolina Research Campus (NCRC), The University of North Carolina at Charlotte, Kannapolis, NC 28081, United States; Department of Bioinformatics and Genomics, Computational Intelligence to Predict Health and Environmental Risks (CIPHER) Research Center, The University of North Carolina at Charlotte, Charlotte, NC 28223, United States; Department of Bioinformatics and Genomics, North Carolina Research Campus (NCRC), The University of North Carolina at Charlotte, Kannapolis, NC 28081, United States; Department of Bioinformatics and Genomics, Computational Intelligence to Predict Health and Environmental Risks (CIPHER) Research Center, The University of North Carolina at Charlotte, Charlotte, NC 28223, United States; Department of Bioinformatics and Genomics, North Carolina Research Campus (NCRC), The University of North Carolina at Charlotte, Kannapolis, NC 28081, United States; Department of Bioinformatics and Genomics, Computational Intelligence to Predict Health and Environmental Risks (CIPHER) Research Center, The University of North Carolina at Charlotte, Charlotte, NC 28223, United States

## Abstract

**Motivation:**

Polymerase chain reaction (PCR) is the world’s most important molecular diagnostic with applications ranging from medicine to ecology. PCR can fail because of poor primer design. The nearest-neighbor thermodynamic properties, picking conserved regions, and filtration via penalty of oligonucleotides form the basis for good primer design.

**Results:**

DeGenPrime is a console-based high-quality PCR primer design tool that can utilize MSA formats and degenerate bases expanding the target range for a single primer set. Our software utilizes thermodynamic properties, filtration metrics, penalty scoring, and conserved region finding of any proposed primer. It has degeneracy, repeated *k*-mers, relative GC content, and temperature range filters. Minimal penalty scoring is included according to secondary structure self-dimerization metrics, GC clamping, tri- and tetra-loop hairpins, and internal repetition. We compared PrimerDesign-M, DegePrime, ConsensusPrimer, and DeGenPrime on acceptable primer yield. PrimerDesign-M, DegePrime, and ConsensusPrimer provided 0%, 11%, and 17% yield, respectively, for the alternative iron nitrogenase (*anfD*) gene target. DeGenPrime successfully identified quality primers within the conserved regions of the T4-like phage major capsid protein (*g23*), conserved regions of molybdenum-based nitrogenase (*nif*), and its alternatives vanadium (*vnf*) and iron (*anf*) nitrogenase. DeGenPrime provides a universal and scalable primer design tool for the entire tree of life.

**Availability and implementation:**

DeGenPrime is written in C++ and distributed under a BSD-3-Clause license. The source code for DeGenPrime is freely available on www.github.com/raw-lab/degenprime.

## 1 Introduction

Polymerase chain reaction (PCR) is the most important fundamental tool for molecular diagnostics, genetic analysis, viral load testing, molecular biology, phylogenetics, and a plethora of other disciplines (Yang and Rothman 2004, Filée *et al.* 2005, Lorenz 2012, White III 2021). A major cause of failure in PCR experiments is a poor choice in primers. Rules of good design for PCR primers are well-established; they should be 15–30 bp long, without complementary ends, contain between 40% and 60% guanine (G) or cytosine (C), have minimal dinucleotide repeats, and similar melting temperatures (Lorenz 2012, Sambo *et al.* 2018). Primer3 is the gold-standard tool for finding excellent candidate primers for single gene sequences (Koressaar and Remm 2007, Untergasser *et al.* 2012). This tool was not designed to find primers for the multi-sequence alignments (MSAs) often used in phylogenetic studies and does not support the addition of degenerate bases.

When comparing closely related species, there will be some conserved regions of DNA (Frazer *et al.* 2003). In fact, as two species are more distantly related, the conserved regions tend to disappear (Tamames 2001). Conserved regions are the optimal locations for PCR experiments as there can be no primer matching bias or mismatches when the target region is identical across all sequences (Sambo *et al.* 2018).

DeGenPrime aims to find primers for MSAs based on the conserved regions found across the sequences without relying on any reference, instead using the general principles of good primer design. Beyond a conserved region approach, DeGenPrime also utilizes a filtration digital module that hard filters primers based on limited degeneracy. Our software can build primers independently without bias in a highly scalable manner resolving the diverse and continuously evolving biosphere.

## 2 Program design and methods

**Figure 1.**
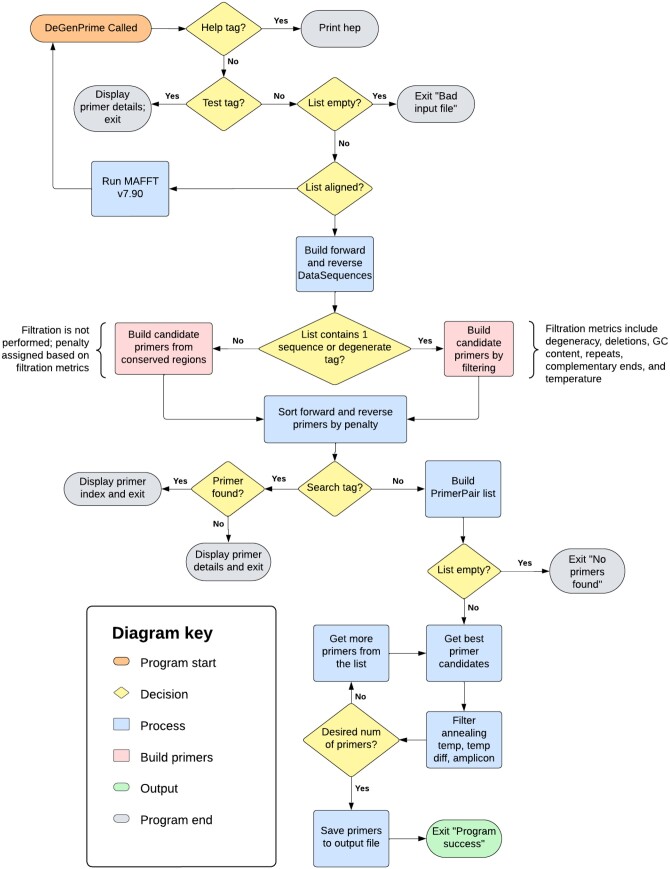
Flowgram of the DeGenPrime software. It can utilize a single sequence or a MSA as input for primer design. Input formats include a nucleotide or protein single fasta file or a clustal MSA format. It provides two major paths for primer selection including a filtration versus conserved region approach for primer design. DeGenPrime minimizes degeneracy but higher degeneracy can we requested upon user parameters. Outputs provided is a list of primers in.dgp format, scoring metrics, filtration results, and consensus sequence.

DeGenPrime provides an overall tool kit for primer design via filtration or conserved region picking (Fig. 1). DeGenPrime input formats include a nucleotide fasta file or a nucleotide MSA with preprocessor tags in clustal format. User tags are processed and stored as static global variables for access throughout various parts of the program. After loading input data, the program checks the sequence file for selected format either a collection of sequences that aren’t aligned, a previously aligned collection of sequences, or a single gene sequence. The quality controls list whether the sequence file is correctly formatted, aligned, and/or an empty file. If it is not aligned, the program aligns the sequences via MAFFT (multiple alignment fast Fourier transform) either using global or local based on user specifications with a maximum iteration of 1000 (Katoh and Standley 2013).

After these basic formatting checks are complete, DeGenPrime restructures the list of sequences into two lists of nodes representing the forward and reverse DNA sequences. Each node in this new list represents what is happening at each individual base pair location across all the sequences. It selects the most common nucleotide letter from the bp location, finds the ratio of this letter out of all possible nucleotides (A, T, G, C), and based on that ratio determines if a degenerate nucleotide code should be chosen. After this the program begins scanning the restructured list for regions where no degenerate nucleotides were identified. Regions large enough to accommodate a primer are considered conserved regions. This is our conserved region approach for MSA files by default (Fig. 1). Once all of the conserved regions have been identified, the program runs a quick check to make sure that there are enough conserved regions or a single large conserved region able to produce a large enough amplicon (based on user specifications) for forward and reverse primers. If there are insufficient regions to find conserved primers, then the program will show the user the consensus sequence and abort. Otherwise, the possible primers within the conserved regions will be calculated and scored by primer calculator objects.

For single sequences or if the user wants some limited degeneracy, DeGenPrime can use a filtering process to generate primers instead of the conserved region approach (Fig. 1). If a user specifies a single sequence only the filter module will be used exclusively. Forward and reverse primer calculator objects are constructed which contain lists of all possible forward and reverse primers based on the user defined parameters of if they want a minimum amplicon size or a specified range of base pairs (measuring bp 0 from the 5′ end onward). The primer calculators have built in filters for the primer lists that limit their degeneracy, deletions, GC ratio, internal repetition, melting temperature and complementary ends. Lists are filtered for primers until only the best primers remain.

The degeneracy filter is a novelty of this program. It measures the degeneracy of a single individual bp location providing the best possible base whether degenerate or not. Degenerate codes within the first or last three nucleotides within a primer will disqualify it. If any “N” base is found, it is labeled as too degenerate which disqualifies a primer because it’s a 4-fold degenerate base (A, T, C, G all possible). We further limit a primer to having only one 3-fold degenerate base (e.g. H, B, V, D), and up to two 2-fold degenerate bases (e.g. S, R, Y, M, K, W) per primer. Deletions are especially problematic in primer selection and design. We designed a deletion filter that removes primers based on these rules: (i) if deletions occur within the first or last three nucleotides, (ii) there are more than three consecutive deletions, (iii) more than six total deletions are found, and/or (iv) when the deletion causes the primer to drop below the minimum size threshold (e.g. <18 bp).

GC content must be accounted for in robust primer design. Having on average 40%–60% composition per primer is required for proper Tm for the primer set. Also, having no more than 3 G or C nucleotides at the 3′ end of the primer to promote binding at annealing steps but to also limit dimerization. Our GC content filter restricts all primers regarding these parameters.

Repetitions in primers are challenging including reducing sensitivity and specificity (Hommelsheim *et al.* 2014). Amplification of repetitive DNA can increase chimeras and artifacts (Hommelsheim *et al.* 2014). We included a primer repetition filter based on *k-*mer counting and matching for length *K *=* *2, 3, or 4 nucleotide matches that exist within the primer itself. Primers with higher matches of the variable length *K* within the filter will be excluded due to the likelihood of primer misbinding. The default for DeGenPrime based on our *k*-mer filter will allow for two dinucleotides, a single trinucleotide, and no tetranucleotide matches. For program efficiency, DeGenPrime does not check *k-*mers larger than four nucleotides long.

Complementary ends enhance dimer formation during PCR; these must be discarded. We use a 3-mer based filter that checks the last three nucleotides of a primer for complementary ends. If a complementary match occurs within the primer, it is disqualified.

Hairpins and dimers penalty score is calculated for all sequences that have the greatest likeliness to form triloop and tetraloop hairpins (Lu *et al.* 2006). Triloop hairpins occur often in a 5-bp track, where the first and last bp are complementary, and when the second bp position and the second to last bp position are G and A, respectively (SantaLucia and Hicks 2004). Tetraloop hairpin follows a similar pattern as the triloop but on a 6-bp track over a 5-bp track that is complementary. Furthermore, with our previous filter limits the candidate primers in the list to contain between 40% and 60% GC content, there is an above average probability of having a high GC-content 5-mer with too negative delta G (e.g. GGTGG) and is also complementary to another part of the primer. Dimerization scoring takes the last 5-mer of the primer then measures the Gibbs free energy (Desmarais *et al.* 2012). The nearest-neighbor formula for Gibbs free energy is:

(1) $$\Delta G{}^{\circ}\left(\mathrm{total}\right)=\Sigma_{i}n_{i}\Delta G{}^{\circ}\left(i\right)+\Delta G{}^{\circ}\left(\mathrm{init}w/\mathrm{term}G/C\right)+\Delta G{}^{\circ}\left(\mathrm{init}w/\mathrm{term}A/T\right)+\Delta G{}^{\circ}\left(\mathrm{sym}\right)$$

See SantaLucia (1998) for equation details.

While these hairpins and dimers are not excluded from DeGenprime they are ranked lower via penalty scoring. We use a lambda function to resolve the sorting based on the combined hairpin/dimer filter which adds a penalty to all primers with Gibbs free energy less than −3 kcal/mol or if internal nucleotides match either of these patterns.

DeGenprime does thermodynamic calculations based on our current defaults for temperature, ion concentration, and primer concentration. All primers by default range from 50°C to 65°C melt temperature, 50 mM monovalent ion concentration, and 50 nM concentration of the primer itself. However, the user can specify a narrower temperature range or a selected range within our global settings. It then uses thermodynamic calculations based on the nearest-neighbor formula to determine the melting temperature of the primer. The formula is given by:

(2) $$T_{m}=\frac{\Sigma \left(\Delta H_{d}\right)+\Delta H_{i}}{\Sigma \left(\Delta S_{d}\right)+\Delta S_{i}+\Delta S_{\mathrm{self}}+R\;\ln\left(\frac{C_{T}}{b}\right)}+16.6\log[{\mathrm{Na}}^{+}]$$

See Panjkovich and Melo (2005) for equation details. Penalty is added to any primer whose melting temperature falls below the minimum or above the maximum temperature.

Within DeGenPrime, we first apply filters and scoring metrics to primer list sets (i.e. forward and reverse) independently of the primer pairing. After independent filtering and scoring of forward and reverse primers, we produce sorted lists of primers with the minimum accrued penalty first. The program then checks to see if primers were present; if not this provides an error message of “no primer found,” to the user. After which we apply filtering/scoring to primer pairings.

**Figure 2.**
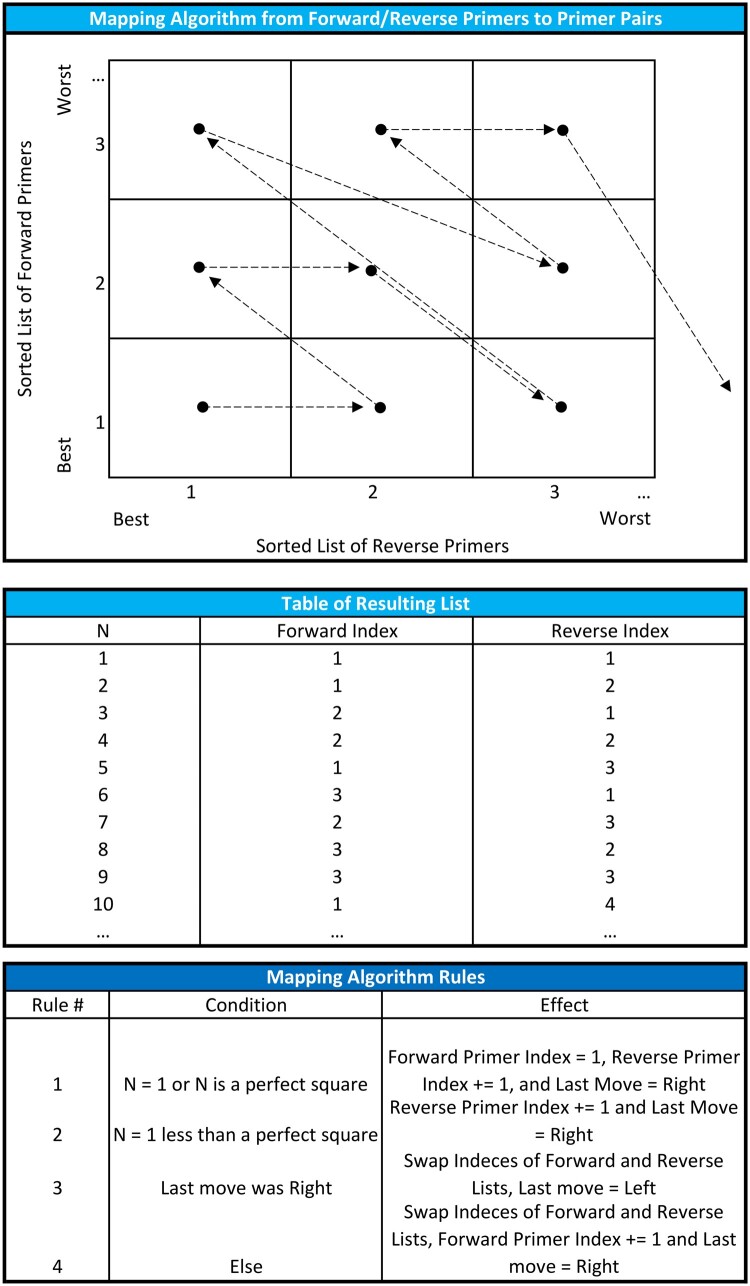
Mapping algorithm diagram. DeGenPrime utilizes a greedy loop algorithm that rank choices via step-wise mapping applying a systematic quality-control and efficient sorting of ranked lists for primer-pair optimization.

Building a list of primer pairings and several of the filtering operations on these massive forward and reverse primer lists results in slow operations and an *O*(*n*^2^) problem. To make the program run more efficiently, we created a mapping algorithm for primer pairing (Fig. 2). This algorithm partitions the data to ∼1600 pairs per block, then examines them one block at a time, then moves to the next block searching for optimal primer pairs exhaustively. The program loops through until it finds five highly optimized primer pairs or results in none found. While the block size (i.e. 1600) is arbitrary; it empirically provides a reasonable transition speed at runtime. We filter the partitions via a minimum amplicon (i.e. 100 bp default), a *T*_m_ with 1°C between primer pairs, and annealing temperature between the pairs that are <5°C. We calculate annealing temperature of the primer pair using this formula:

(3) $$T_{m}\left(\mathrm{product}\right)=0.41\left(\%G+\%C\right)+16.6\log[K^{+}]\;-\;\frac{675}{l}$$

See Rychlik *et al.* (1990) for equation details. If no primers are found, the user is notified. Otherwise, the final list of primer pairs is outputted to the user.

As a generalizable feature of DeGenPrime, we offer a primer testing module, to test previously assembled and designed primers or their pairs. The user can directly enter a candidate primer, then DeGenPrime performs calculations based on the aforementioned nearest neighbor formula (Equation 2), and lists whether the primer will pass various filters.

Another feature of DeGenPrime is the primer searching module. This module allows users to search for specific forward and reverse primers within their respective candidate primer lists. The program will scan each respective list for the candidate primer and alert the user if that primer was found or not found. If the primer was not found, the candidate primer is pipelined into the testing module, giving the user a clear picture of why this primer failed. Note the primer is not guaranteed to match any region within the MSA or sequence.

## 3 Results and discussion

The novel nature of DeGenPrime makes it difficult to compare to other software. Primer3 is the top primer tool available, but its results cannot be used as a fair basis of comparison. Primer3 is only designed to process individual sequences and cannot be applied with MSA formats or support for degenerate bases like DeGenPrime. We compared Primer3 to DeGenPrime directly, finding a comparable runtime, similar penalty scoring metrics, and similar primers across both tools when utilizing one gene sequence.

EcoFunPrimer and its metagenomic version MetaFunPrimer offers an MSA processing functionality (Liu *et al.* 2021). Currently, EcoFunPrimer and MetaFunPrimer cannot install, has run time errors, doesn’t compile, and currently is no longer supported. HYDEN is another and one of the first degenerate primer design softwares; however, the last update was in 2008, and it is only available in a depreciated Windows XP (Linhart and Shamir 2007).

Some approaches to MSA primer design, like the JCVI primer designer, often make use of reference sequences (Li *et al.* 2008, 2012). One problem with this approach is reference sequences introduce a primer matching bias within interspecies comparisons favoring sequences with the most matches to the given reference (Huszar *et al.* 2019). Within the human microbiome, where intraspecies genetic diversity and mutation rates may be higher, the reference sequence may not remain a valid basis of comparison for any particular experiment (Ramiro *et al.* 2020). The JCVI program is no longer in active development, with the last update in 2013, and DeGenPrime is independent of a reference sequence.

We tested our program against PrimerDesign-M, which often makes use of reference sequences (Yoon and Leitner 2015). Currently, PrimerDesign-M is only available by web browser, not a standalone software. We utilized our primer testing module on the output from PrimerDesign-M, for iron nitrogenase (*anfD*) and found 100% of the primers failed our filters, due to including too much degeneracy (Table 1), and 90% failed due to having too much GC Content which shows this approach also does not consider basic primer quality filtering.

**Table 1.** PrimerDesign-M target yield for alternative nitrogenase (*anfD*).

Set	Primer codes	Orientation	Found?	Filters failed
1	WGCACGCCGTGRTSAAGGGC	Forward	No	Degeneracy, GC content
	TGCCAGGTGTCGTAGGTGCAGCCS	Reverse	No	Degeneracy, GC content, Temp
2	WGCACGCCGTGRTSAAGGGC	Forward	No	Degeneracy, GC content
	GCCAGGTGTCGTAGGTGCAGCCS	Reverse	No	Degeneracy, GC content, Temp
3	WGCACGCCGTGRTSAAGGGC	Forward	No	Degeneracy, GC content
	CCAGGTGTCGTAGGTGCAGCCS	Reverse	No	Degeneracy, GC content
4	WGCACGCCGTGRTSAAGGGC	Forward	No	Degeneracy, GC content
	CAGGTGTCGTAGGTGCAGCCS	Reverse	No	Degeneracy, GC content
5	WGCACGCCGTGRTSAAGGGC	Forward	No	Degeneracy, GC content
	AGGTGTCGTAGGTGCAGCCS	Reverse	No	Degeneracy, GC content
**Summary**				
**Primer was found:**		0		0%
**Primer was not found:**		10		100%
**Total:**		10		100%

PrimerDesign-M was ran using default parameters.

The similarly named perl program called Degeprime, provided another comparison to DeGenPrime (Hugerth *et al.* 2014). Directly compared both programs using nitrogenase and T4-like major capsid protein (*g*23) MSA as input. We applied a search function for this direct comparison analyzing the top primers between Degeprime as a sorted list of primers. First Degeprime doesn’t report primer pairs, only forward primers making comparisons challenging. However, we scored the forward primers provided by Degeprime, ∼11% of the forward primers passed (Table 2), but no reverse primers, thus not a generalizable tool for robust primer design. Degeprime does not include any filtering for melting temperature ranges, GC content or complementary ends; all of which are basic criteria for making a good primer. Furthermore, some of the primers it suggested which are theoretically good primers were not located within the conserved region of the MSA. Degeprime seems to be just printing a list of all possibilities of primers without considering any quality standards.

**Table 2.** DegePrime target yield for phage major capsid (*g23*), nitrogenase (*nifDK*), and alternative nitrogenases (*anfDK/vnfDK*).

DegePrime output versus DeGenPrime filters					
Data	Num	DeGenPrime primer	Found?	Rank	Filters failed
***anfD***	1	GTGCAGCGAGTGCATCCCGG	No		Temp, GC%
	2	TGCAGCGAGTGCATCCCGGA	No		Temp, GC%
	3	GCAGCGAGTGCATCCCGGAG	No		Temp, GC%
	4	CAGCGAGTGCATCCCGGAGC	No		Temp, GC%
	5	AGCAAGTGCATCCCGGAGCG	Yes	749	
	6	GCGAGTGCATCCCGGAGCGC	No		Temp, GC%, comp. ends
	7	CAAGTGCATCCCGGAGCGCA	Yes	751	
	8	AAGTGCATCCCGGAGCGCAA	Yes	752	
	9	AGTGCATCCCGGAGCGCAAG	Yes	753	
	10	GTGCATCCCGGAGCGCAAGA	Yes	754	
***anfK***	1	GAAGGAGCGCATCGGCACCA	No		Temp, GC%
	2	AAGGACCGCGTGGGCACCAT	No		Temp, GC%
	3	AGGACCGCGTGGGCACCATC	No		Temp, GC%
	4	CGAGCGCCCGGGCATCATCA	No		Temp, GC%
	5	GCCCGCGCCGGCGTGATCAA	No		Temp, GC%
	6	GCCGCGTGGGCACCATCAAC	No		Temp, GC%
	7	CCGCGTGGGCACCATCAACC	No		Temp, GC%
	8	CGCGTGGGCACCATCAACCC	No		Temp, GC%
	9	GCGTGGGCACCATCAACCCG	No		Temp, GC%
	10	CGTGGGCACCATCAACCCGA	No		GC%
***g23***	1	CTGGTCCTACTGGACTGATC	No		
	2	TGGTCCTACTGGACTGATCT	No		
	3	GGTCCTACTGGACTGATCTT	No		Temp
	4	GTCCTACTGGACTGATCTTC	No		Temp
	5	TCCTACTGGACTGATCTTCG	No		
	6	CCTACTGGACTGATCTTCGC	No		
	7	CTACTGGACTGATCTTCGCA	No		
	8	TACTGGACTGATCTTCGCAA	No		
	9	ACTGGACTGATCTTCGCAAT	No		
	10	CTGGACTGATCTTCGCAATG	No		
***nifD***	1	CTGCGACAAGCCGATCCCGG	No		Temp, GC%
	2	TGCGACAAGCCGATCCCGGA	No		Temp, GC%
	3	GCGACAAGCCGATCCCGGAG	No		GC%
	4	CGACAAGCCGATCCCGGAGC	No		GC%
	5	GACAAGCCGATCCCGGAGCG	No		GC%
	6	ACAAGCCGATCCCGGAGCGC	No		Temp, GC%
	7	CAAGCCGATCCCGGAGCGCA	No		Temp, GC%
	8	AAGCCGATCCCGGAGCGCAT	No		Temp, GC%
	9	AGCCGATCCCGGAGCGCATG	No		Temp, GC%
	10	GCCGATCCCGGAGCGCATGA	No		Temp, GC%
***nifK***	1	CCGCGAGGCCCTGACCGTGA	No		Temp, GC%
	2	CGCGAGGCCCTGACCGTGAA	No		Temp, GC%
	3	GCGAGGCCCTGACCGTGAAC	No		Temp, GC%
	4	CGAGGCCCTGACCGTGAACC	No		GC%
	5	GAGGCCCTGACCGTGAACCC	No		GC%
	6	AGGCCCTGACCATCAACCCG	No		GC%
	7	GGCCCTGACCGTGAACCCGG	No		Temp, GC%
	8	GCCCTGACCATCAACCCGGC	No		Temp, GC%, comp. ends
	9	CCCTGACCATCAACCCGGCC	No		GC%
	10	CCTGACCATCAACCCGGCCA	No		GC%
***vnfD***	1	GTGCGACAAGGACATCCCGG	No		GC%
	2	TGCGACAAGGACATCCCGGA	No		GC%
	3	GCGACGAGACCATCCCGGAG	No		GC%
	4	CGACGAGACCATCCCGGAGC	No		GC%
	5	GACGAGACCATCCCGGAGCG	No		GC%
	6	ACGAGACCATCCCGGAGCGC	No		Temp, GC%
	7	CAAGGACATCCCGGAGCGCG	No		Temp, GC%
	8	AAGGACATCCCGGAGCGCCA	No		Temp, GC%
	9	AGGACATCCCGGAGCGCGAG	No		Temp, GC%
	10	GGACATCCCGGAGCGCCAGA	No		Temp, GC%
***vnfK***	1	CAAGGACCGCGCCGGCATCA	No		Temp, GC%
	2	AAGGACCGCGCCGGCATCAT	No		Temp, GC%
	3	AGGACCGCGCCGGCATCATC	No		Temp, GC%
	4	GGACCGCGCCGGCATCATCA	No		Temp, GC%
	5	GACCGCGCCGGCATCATCAA	No		Temp, GC%
	6	ACCGCGCCGGCATCATCAAC	No		Temp, GC%
	7	CCGCGCCGGCATCATCAACC	No		Temp, GC%
	8	CGCGCCGGCATCATCAACCC	Yes	13	
	9	GCGCCGGCATCATCAACCCG	Yes	14	
	10	CGCCGGCATCATCAACCCGA	Yes	15	
**Summary**					
**Primer was found:**			8		11%
**Primer was not found:**			62		89%
**Total:**			70		100%

DegePrime was ran using default parameters.

ConsensusPrimer is a program which, like ours, finds the conserved regions of its MSA to build its candidate list of primers (Collatz *et al.* 2022). It calls MAFFT to make its alignment and runs Primer3 on this alignment to find its primers. We ran each of our test MSAs into its pipeline and found subtle differences in this approach and ours. ConsensusPrimer was unable to suggest any primers for iron-based nitrogenase (*anfK*), T4-like major capsid protein (*g23*), molybdenum-based nitrogenase (*nifD*), and vanadium-based nitrogenase (*vnfK*) alignments. The primers it did suggest for iron-based (*anfD*) and molybdenum-based (*nifK*) were slightly outside the acceptable range of DeGenPrime’s temperature filter (Table 3). For vanadium-based (*vnfD*), all of the reverse primers were 17 bp long which is less than the size minimum for DeGenPrime, but all of the forward primers passed filter checks. In total, 17% of primers found through this pipeline were acceptable via DeGenPrime standards, however, many of the primer pairings suggested had a forward or reverse primer repeatedly showing up in other pairings, while DeGenPrime only outputs unique primers.

**Table 3.** ConsensusPrimer primers evaluated using DeGenPrime (test mode) for phage major capsid (*g23*), nitrogenase (*nifDK*), and alternative nitrogenases (*anfDK/vnfDK*).

Data	Num	ConsensusPrimer primer	Found?	Rank	Filters failed
***anfD***	1	CTTCCAGCTGAAGTACACC	No		Temp
	2	CACCACAAGATCAACATCG	No		Temp
	3	TTCCAGCTGAAGTACACC	No		Temp
	4	CACCACAAGATCAACATCG	No		Temp
	5	CTTCCAGCTGAAGTACACC	No		Temp
	6	CCACCACAAGATCAACATC	No		Temp
	7	TTCCAGCTGAAGTACACC	No		Temp
	8	CCACCACAAGATCAACATC	No		Temp
	9	TTCCAGCTGAAGTACACCT	No		Temp
	10	CACCACAAGATCAACATCG	No		Temp
***nifK***	1	TGAAGACCAGCATCAAGAA	No		Temp
	2	AACAACAAGGTGAACCTGAT	No		Temp
	3	TGAAGACCAGCATCAAGAA	No		Temp
	4	ACAACAAGGTGAACCTGAT	No		Temp
	5	TGAAGACCAGCATCAAGAA	No		Temp
	6	CTTCAGCAACATGGTGAAG	No		Temp, comp. ends
	7	GAAGACCAGCATCAAGAAC	No		Temp
	8	AACAACAAGGTGAACCTGAT	No		Temp
	9	CTGAAGACCAGCATCAAGAA	No		Temp
	10	AACAACAAGGTGAACCTGAT	No		Temp
***vnfD***	1	GGACTTCGAGAAGGTGATC	Yes	4902	
	2	GTACATGGGCTTCGAGG	No		Size
	3	AGGACTTCGAGAAGGTGAT	Yes	4901	
	4	GTACATGGGCTTCGAGG	No		Size
	5	GAGGACTTCGAGAAGGTG	Yes	4700	
	6	GTACATGGGCTTCGAGG	No		Size
	7	GACTTCGAGAAGGTGATCG	Yes	4903	
	8	GTACATGGGCTTCGAGG	No		Size
	9	AACGAGCTGGAGTTCTTC	Yes	6113	
	10	GTACATGGGCTTCGAGG	No		Size
**Summary**					
**Primer was found:**			5		17%
**Primer was not found:**			25		83%
**Total:**			30		100%

ConsensusPrimer was ran using default parameters. Genes *anfK*, *g23*, *nifD*, and *vnfK* yielded no primers.

A last testing method was applied to this program to see if it could produce primer pairs used in another experiment. One such experiment found degenerate primers for the T4-like major capsid protein (*g23*) phages (Filée *et al.* 2005). Using the public data provided from this experiment, DeGenPrime produced forward and reverse primers which overlapped the primers used in this experiment (Table 4).

**Table 4.** DeGenPrime yield for phage major capsid (*g23*), nitrogenase (*nifDK*), and alternative nitrogenases (*anfDK/vnfDK*).

Data	Pair	Forward primer	Index	Length	Reverse primer	Index	Length
***anfD***	1	AGTTCGAGTGCAGCAAGT	13	18	CTGCTTCAGCAGCTTCTC	350	18
	2	TTCGAGTGCAGCAAGTGC	15	18	TGCTTCAGCAGCTTCTCG	349	18
	3	GTTCGAGTGCAGCAAGTG	14	18	GCCTCGATGATGTTCTGC	364	18
	4	ATGCCGTACCACGAGTTC	0	18	CCTTGAAGGCCTCGATGA	372	18
	5	TGCCGTACCACGAGTTCG	1	18	GGCCTTGAAGGCCTCGAT	374	18
***anfK***	1	CTGCGAGGTGAAGGAGAAGG	5	20	CAGGCGCACGAACATCAC	152	18
	2	TCTTCACCTGCCAGCCGG	49	18	AGCAGGCGCACGAACATC	154	18
	3	TGCGAGGTGAAGGAGAAGG	6	19	GAACATCACGCAGCCCTG	143	19
	4	ATCTTCACCTGCCAGCCG	48	18	CGCACGAACATCACGCAG	148	18
	5	CCCGATCTTCACCTGCCA	44	18	CACGAACATCACGCAGCC	146	18
***g23***	1	GGGTTCAGCCGATGACTG	347	18	GCGGTTGATTTCCAGCATG	1190	19
	2	GGGGTTCAGCCGATGACTG	346	19	CGCGGTTGATTTCCAGCATG	1191	20
	3	GATATTTGGGGGGTTCAGC	337	19	CGGTTGATTTCCAGCATGAT	1189	20
	4	ATATTTGGGGGGTTCAGC	338	18	GGTTGATTTCCAGCATGAT	1188	19
***nifD***	1	CGCGGCTGCGCCTACGCC	294	18	GCCCCAGCTGTAGTAGCCGCAGC	398	23
	2	GCGGCTGCGCCTACGCCG	295	18	CCCCAGCTGTAGTAGCCGCAGCC	397	23
	3	TGGGCTGCGGCTACTACA	373	18	ATGTCGTCGCCGATCAGG	373	18
	4	GGGCCCGATCAAGGACAT	332	18	GATGTCGTCGCCGATCAG	635	18
	5	GGCTGCGGCTACTACAGC	375	18	CGATGTCGTCGCCGATCA	636	18
***nifK***	1	TCGTGCACGGCAGCCAGG	307	18	AACACGGCGGCGTCCTCG	418	18
	2	TGCACGGCAGCCAGGGCT	310	18	CCGAACACGGCGGCGTCCT	421	19
	3	GCACGGCAGCCAGGGCTG	311	18	CGAACACGGCGGCGTCCT	420	18
	4	GTGCACGGCAGCCAGGGCT	309	19	ACACGGCGGCGTCCTCGG	417	18
	5	CGTGCACGGCAGCCAGGGCT	308	20	GCCGAACACGGCGGCGTCC	422	19
***vnfD***	1	GCTGAAGCTGCTGAAGTG	5	18	TGGATGGTGTCCTTCAGC	199	18
	2	CGCTGAAGCTGCTGAAGTG	4	19	CGGGCCGTGGATCATCTG	215	18
	3	ATGCCGCTGAAGCTGCTGAAG	0	21	CAGCACGCCGCCGATCAC	185	18
	4	CCGCTGAAGCTGCTGAAGTG	3	20	GGTGTCCTTCAGCACGCC	194	18
	5	TGCCGCTGAAGCTGCTGAAG	1	20	CTTCAGCACGCCGCCGAT	188	18
***vnfK***	1	GGCATCATCAACCCGATG	66	18	CTGGCGATGTCGAAGTTC	226	18
	2	CGGCATCATCAACCCGATG	65	19	GCTGGCGATGTCGAAGTT	227	18
	3	CCGGCATCATCAACCCGA	64	18	TGCTGGCGATGTCGAAGT	228	18

DeGenPrime was run using the conserved region approach using default.

We evaluated 72 16S small subunit ribosomal RNA primer sets that had *in vitro* measurements of PCR from a previous study (Kayama *et al.* 2021). The primers were tested against 31 diverse templates that had ranges of amplification with some working on numerous templates and others that could amplify a few templates but not all. To better evaluate, we focused on primer sets that always failed to amplify templates; these are primer sets 7, 20–22, 46, 49, 55, 60, 68, 71 (Supplementary Table S1). DeGenPrime running in the evaluate primer module agreed that 7 out of the 10 primer sets would fail outright (Supplementary Table S1). DeGenPrime found no reason with its current test primer evaluation module to fail primer set 46, 68, 71 which failed *in vitro* PCR (Supplementary Table S1). However, we do not have the original target sequence(s) or consensus alignment for primer set 46, 68, and 71 to evaluate whether our consensus primer selection approach would fail these primers based on other criteria. DeGenPrime evaluated all primer sets within the Kayama *et al.* (2021). manuscript; however, it is unable to find primers that work 5%–50% of the time *in silico* but future versions of DeGenPrime will include approaches such as recurrent neural networks that may approve this.

We directly compared outputs from Primer3 using the *Azotobacter vinelandii* strain DJ *nifD* gene (GenBank: CP001157.1_137758-139236) and *nifH* gene (GenBank: CP001157.1_136759-137631) for four primer sets each with variable amplicon size providing the best predicted primer set. DeGenPrime test primer evaluation module found that two of the primer sets failed due to end GC content at 80% (Supplementary Table S2). All the primer sets had no degenerate bases satisfying the main goal of DeGenPrime to minimize such bases.

## 4 Conclusions

The importance of having robust and accurate primer design provides time saving and financial ease for molecular diagnostics. DeGenPrime provides a novel, robust, effective approach while concurrently providing a primer evaluator. Due to the novel design of DeGenPrime it can expand the gene target amplification range of the primers which provides more targets per PCR primer set. We elucidate primer pairs while avoiding a majority of the degenerate base pairing issues. Our filtering and conversed region approaches allow for rapid primer discovery for a variety of fields within biology. Both a GUI module and web-based versions are also currently in development providing greater accessibility to the community at large.

DeGenPrime directly evaluates whether primers are high quality or not via a variety of methods mentioned previously. Novel approaches such as machine and/or deep learning may approve our evaluation of primers and their design in the future (Kayama *et al.* 2021). We may include such models such as recurrent or convolutional neural network approaches to DeGenPrime in the future in order to train our evaluator on both filtering and quality metrics. DeGenPrime illuminates primer design unlocking the dark matter within the tree of life.

## Supplementary Material

vbae044_Supplementary_Data

## Data Availability

Raw files, code. supplemental data, and source codes, are all available on www.github.com/raw-lab/DeGenPrime.

## References

[vbae044-B1] Collatz M , Braun SD, Monecke S et al ConsensusPrime—a bioinformatic pipeline for ideal consensus primer design. BioMedInformatics 2022;2:637–42.

[vbae044-B2] Desmarais SM , Leitner T, Barron AE et al Quantitative experimental determination of primer-dimer formation risk by free-solution conjugate electrophoresis. Electrophoresis 2012;33:483–91. 22331820 10.1002/elps.201100452 PMC3416024

[vbae044-B3] Filée J , Tétart F, Suttle CA et al Marine T4-type bacteriophages, a ubiquitous component of the dark matter of the biosphere. Proc Natl Acad Sci USA 2005;102:12471–6. 16116082 10.1073/pnas.0503404102 PMC1194919

[vbae044-B4] Frazer KA , Elnitski L, Church DM et al Cross-species sequence comparisons: a review of methods and available resources. Genome Res 2003;13:1–12. 12529301 10.1101/gr.222003 PMC430969

[vbae044-B5] Hommelsheim CM , Frantzeskakis L, Huang M et al PCR amplification of repetitive DNA: a limitation to genome editing technologies and many other applications. Sci Rep 2014;4:5052. 24852006 10.1038/srep05052 PMC4031481

[vbae044-B6] Hugerth LW , Wefer HA, Lundin S et al DegePrime, a program for degenerate primer design for broad-taxonomic-range PCR in microbial ecology studies. Appl Environ Microbiol 2014;80:5116–23. 24928874 10.1128/AEM.01403-14 PMC4135748

[vbae044-B7] Huszar TI , Wetton JH, Jobling MA et al Mitigating the effects of reference sequence bias in single-multiplex massively parallel sequencing of the mitochondrial DNA control region. Forensic Sci Int Genet 2019;40:9–17. 30682697 10.1016/j.fsigen.2019.01.008 PMC6461131

[vbae044-B8] Kayama K , Kanno M, Chisaki N et al Prediction of PCR amplification from primer and template sequences using recurrent neural network. Sci Rep 2021;11:7493. 33820936 10.1038/s41598-021-86357-1 PMC8021588

[vbae044-B9] Katoh K , Standley DM. MAFFT multiple sequence alignment software version 7: improvements in performance and usability. Mol Biol Evol 2013;30:772–80. 23329690 10.1093/molbev/mst010 PMC3603318

[vbae044-B10] Koressaar T , Remm M. Enhancements and modifications of primer design program Primer3. Bioinformatics 2007;23:1289–91. 17379693 10.1093/bioinformatics/btm091

[vbae044-B11] Linhart C , Shamir R. Degenerate primer design: theoretical analysis and the HYDEN program. Methods Mol Biol 2007;402:221–44. 17951798 10.1007/978-1-59745-528-2_11

[vbae044-B12] Li K , Brownley A, Stockwell TB et al Novel computational methods for increasing PCR primer design effectiveness in directed sequencing. BMC Bioinformatics 2008;9:191. 18405373 10.1186/1471-2105-9-191 PMC2396641

[vbae044-B13] Li K , Shrivastava S, Brownley A et al Automated degenerate PCR primer design for high-throughput sequencing improves efficiency of viral sequencing. Virol J 2012;9:261. 23131097 10.1186/1743-422X-9-261 PMC3548747

[vbae044-B14] Liu J , Villanueva P, Choi J et al MetaFunPrimer: an environment-specific, high-throughput primer design tool for improved quantification of target genes. MSystems 2021;6:e0020121. 34546069 10.1128/mSystems.00201-21 PMC8547451

[vbae044-B15] Lorenz TC. Polymerase chain reaction: basic protocol plus troubleshooting and optimization strategies. J Vis Exp 2012;(63):e3998. 10.3791/3998 PMC4846334 22664923

[vbae044-B16] Lu ZJ , Turner DH, Mathews DH et al A set of nearest neighbor parameters for predicting the enthalpy change of RNA secondary structure formation. Nucleic Acids Res 2006;34:4912–24. 16982646 10.1093/nar/gkl472 PMC1635246

[vbae044-B17] Panjkovich A , Melo F. Comparison of different melting temperature calculation methods for short DNA sequences. Bioinformatics 2005;21:711–22. 15501913 10.1093/bioinformatics/bti066

[vbae044-B18] Ramiro RS , Durão P, Bank C et al Low mutational load and high mutation rate variation in gut commensal bacteria. PLoS Biol 2020;18:e3000617. 32155146 10.1371/journal.pbio.3000617 PMC7064181

[vbae044-B19] Rychlik W , Spencer WJ, Rhoads RE et al Optimization of the annealing temperature for DNA amplification *in vitro*. Nucleic Acids Res 1990;18:6409–12. 2243783 10.1093/nar/18.21.6409 PMC332522

[vbae044-B20] Sambo F , Finotello F, Lavezzo E et al Optimizing PCR primers targeting the bacterial 16S ribosomal RNA gene. BMC Bioinformatics 2018;19:343. 30268091 10.1186/s12859-018-2360-6 PMC6162885

[vbae044-B21] SantaLucia J Jr. , Hicks D. The thermodynamics of DNA structural motifs. Annu Rev Biophys Biomol Struct 2004;33:415–40. 15139820 10.1146/annurev.biophys.32.110601.141800

[vbae044-B22] SantaLucia J. Jr. A unified view of polymer, dumbbell, and oligonucleotide DNA nearest-neighbor thermodynamics. Proc Natl Acad Sci USA 1998;95:1460–5. 9465037 10.1073/pnas.95.4.1460 PMC19045

[vbae044-B151] Tamames J. Evolution of gene order conservation in prokaryotes. Genome Biol 2001;26:RESEARCH0020. 10.1186/gb-2001-2-6-research0020 PMC33396 11423009

[vbae044-B23] Untergasser A, Cutcutache I, Koressaar T et al Primer3—new capabilities and interfaces. Nucleic Acids Res 2012;40:e115. 10.1093/nar/gks596 PMC3424584 22730293

[vbae044-B24] White III RA. The future of virology is synthetic. Msystems 2021;6:e0077021. 34463577 10.1128/mSystems.00770-21 PMC8519122

[vbae044-B25] Yang S , Rothman RE. PCR-based diagnostics for infectious diseases: uses, limitations, and future applications in acute-care settings. Lancet Infect Dis 2004;4:337–48. 15172342 10.1016/S1473-3099(04)01044-8 PMC7106425

[vbae044-B26] Yoon H , Leitner T. PrimerDesign-M: a multiple-alignment based multiple-primer design tool for walking across variable genomes. Bioinformatics 2015;31:1472–4. 25524896 10.1093/bioinformatics/btu832 PMC4410655

